# Supplementary material for: Composite of Layered Double Hydroxide with Casein and Carboxymethylcellulose as a White Pigment for Food Application
Source: Foods. 2022 Apr 13;11(8):1120. doi: 10.3390/foods11081120 (PMC9032268; doi:10.3390/foods11081120)
Supplement: Supplementary file 1 [file foods-11-01120-s001.zip › foods-1621811-supplementary.pdf]

foods MDPI

Supplementary Materials

Composite of layered double hydroxide with casein and carboxymethylcellulose as a white pigment for food application

Estee Ngew, Wut Hmone Phue, Ziruo Liu and Saji George*

Department of Food Science and Agricultural Chemistry, McGill University, Sainte-Anne-de-Bellevue, QC H9X 3V9, Canada
* *Correspondence:* saji.george@mcgill.ca

Matrix Development for Successful Application of synthesized particles compared to commercially used TiO_2 alternatives and E171

The reliability of the particles as white pigments were expressed based on three factors (Efficacy, Cost, and Safety) whereby each factor was given a score distributed between the lowest to the highest values. The details of the scoring system are shown below:

Efficacy: Scattering efficiency is one of the most important criteria of a white pigment, as it relates to the masking ability (tint strength) of the pigment. The average % reflectance at 540-580nm where human eyes are most sensitive was used to portray the efficacy of the particles as white pigments. Based on our experimental results, the points were distributed between 91.91 to 100.13.

Cost: the cost of the commercially utilized white pigments and synthesizing materials of CCLDH were obtained from www.alibaba.com. The material cost of CCLDH includes fixed cost (costs associated with the production of CCLDH) approximately 30% of material base cost.

Table S1. Cost analysis of CCLDH synthesized in this study.

	MgO	Al2O3	NaOH	Casein	CMC	Total
**Cost/ kg (USD)**	0.6	0.46	0.33	2	1.25	
**Mass (kg)**	81.2	102.3		70.9	109.3	363.7
**Cost (USD)**	48.72	47.058	0	141.8	136.625	374.203
**Cost/kg raw LDH (USD)**						1.02888
**Fixed cost (30% of raw LDH) (USD)**						0.308663459
**Cost/kg LDH (USD)**						**1.337541655**

Safety: Biocompatibility is another key element in determining the translation of the pigment from bench to shelf. The No Observed Adverse Effect Level (NOAEL) was used to assess this aspect after a long use. The NOAEL values were obtained from scientific literature, as referenced in the table below:

Table S2. No Observed Adverse Effect Level (NOAEL) of tested materials in this study

Material	NOAEL	Reference
**CCLDH**	12,500-25,000 mg clay/ kg bw per day	[1]
**Starch (E1404-E1452)**	22.1-3,053 mg Starch/ kg bw per day	[2]
**E551**	4,000 – 4,500 mg E551/ kg bw per day	[3]
**E171**	2,250 mg TiO_2/ kg bw per day	[4]

Based on the numbers obtained, the highest concentration were used for those with ranges, and the points were evenly distributed between 2, 250 to 25, 000 mg material/ kg bw per day as displayed on the matrix.

Table S3. Raw data derived from experimental results based on the scoring system.

	CCLDH	Rice starch	E551	E171
**Efficacy (% reflectance)**	100.13	98.65	91.91	99.19
**Safety (NOAEL)**	25,000	5,900	4,500	2,250
**Cost (USD/g)**	1.38	1	3	1.65

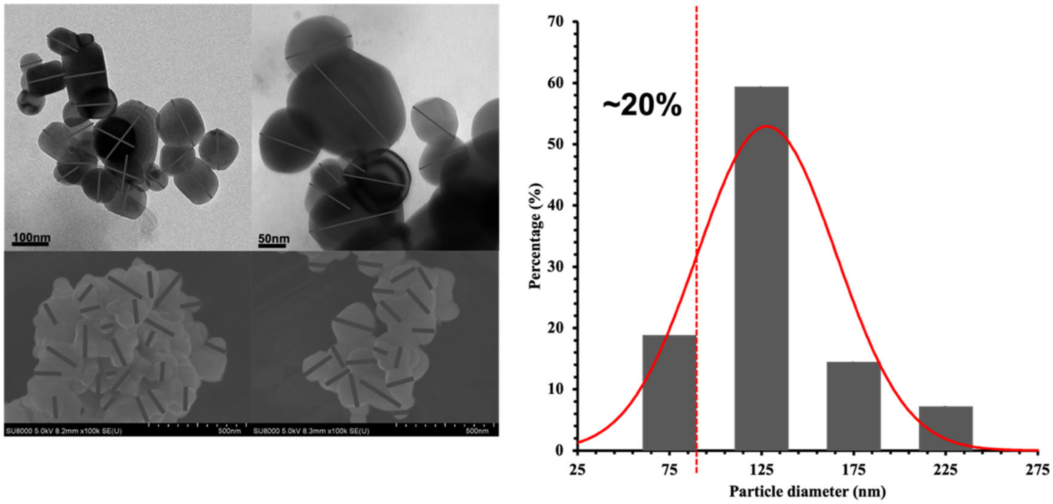

Figure S1. TEM image of E171 used in the study alongside SEM images from our previous studies as a representative sample. The particles were identified by their highly contrast visual with almost spherical shape. The particle mean intensity-based size distribution of E171 particles in diameter (right) analyzed using ImageJ software with the red dotted line separating nanoparticles with their percentage on the left from the larger particles on the right.

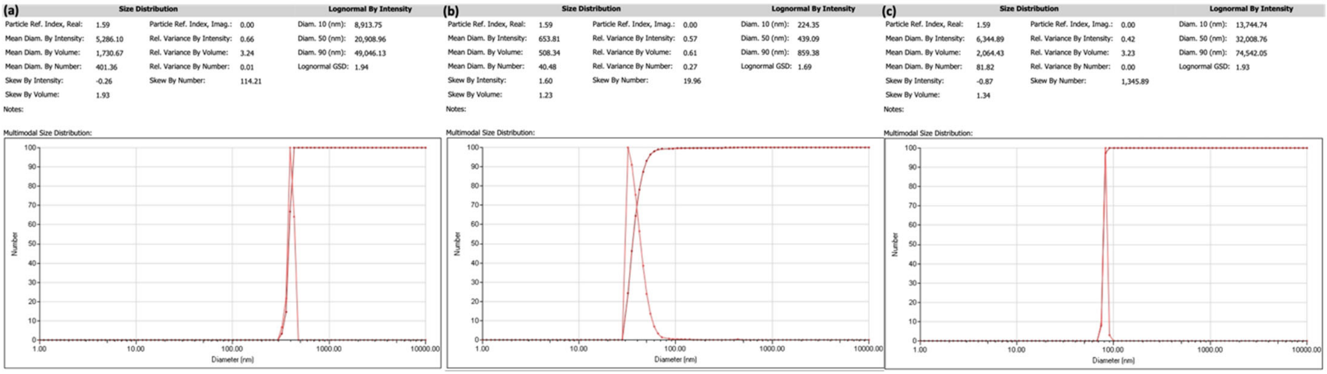

Figure S2. Size distribution analysis of the synthesized particles (a) LDH (b) CLDH and (c) CCLDH.

References

1. SIAM, Organoclays Category, in *SIDS Initial Assessment Profile*. 2007, USA / The Institute of Certified Cost & Management Accountants.
2. Additives, E.Panel o.F., N.S.a.t. Food, A. Mortensen, F. Aguilar, R. Crebelli, A. Di Domenico, B. Dusemund, M.J. Frutos, P. Galtier, D. Gott, et al., Re-evaluation of oxidised starch (E 1404), monostarch phosphate (E 1410), distarch phosphate (E 1412), phosphated distarch phosphate (E 1413), acetylated distarch phosphate (E 1414), acetylated starch (E 1420), acetylated distarch adipate (E 1422), hydroxypropyl starch (E 1440), hydroxypropyl distarch phosphate (E 1442), starch sodium octenyl succinate (E 1450), acetylated oxidised starch (E 1451) and starch aluminium octenyl succinate (E 1452) as food additives. *EFSA Journal*, 2017. **15**(10): p. e04911.
3. Additives, E.Panel o.F., N.S.a.t. Food, M. Younes, P. Aggett, F. Aguilar, R. Crebelli, B. Dusemund, M. Filipič, M.J. Frutos, P. Galtier, et al., Re-evaluation of silicon dioxide (E 551) as a food additive. *EFSA Journal*, 2018. **16**(1): p. e05088.
4. Additives, E.Panel o.F. and N.S.a.t. Food, Re-evaluation of titanium dioxide (E 171) as a food additive. *EFSA Journal*, 2016. **14**(9): p. e04545.
